# Outcomes of a smoking cessation intervention at follow-up after 5 years among tuberculosis patients in China

**DOI:** 10.18332/tid/111539

**Published:** 2019-09-18

**Authors:** Yan Lin, Riitta A. Dlodlo, Qi Shu, Haoxiang Lin, Qin Huang, Xu Meng, Xianglin Zeng, Yongming Chen, Lixin Xiao

**Affiliations:** 1 International Union Against Tuberculosis and Lung Disease, Paris, France; 2 Jinshan District Center for Disease Control and Prevention, Shanghai, China; 3 School of Public Health, Peking University, Beijing, China; 4 Tobacco Medicine and Tobacco Cessation Center, China-Japan Friendship Hospital, Beijing, China; 5 Jiangxi Provincial Institute of Tuberculosis Control and Prevention, Nanchang, China; 6 Ganzhou City Center for Disease Control and Prevention, Ganzhou, China; 7 Ningdu County Tuberculosis Dispensary, Ningdu, China; 8 Xingguo County Tuberculosis Dispensary, Xingguo, China

**Keywords:** smoking cessation, long-term outcome, tuberculosis, China

## Abstract

**INTRODUCTION:**

Smoking cessation should be part of tuberculosis (TB) treatment, but a cessation service is not available as part of a routine TB service in most low- and middle-income countries. WHO and The International Union Against Tuberculosis and Lung Disease (The Union) issued a guideline and China implemented a pilot project 5 years ago. This study aimed to determine changes in smoking status among TB patients at 5 years after completion of anti-TB treatment to observe long-term outcome of a smoking cessation project whose baseline characteristics were associated with a relapse of smoking behavior.

**METHODS:**

A prospective longitudinal study was conducted 5 years after completion of anti-TB treatment to assess changes in patient smoking status against individual baseline data that were entered into a database at the time of TB registration. The patients were tracked by trained village doctors and validated by township health staff. Their smoking status was assessed and entered into the database and analysed.

**RESULTS:**

Of the 800 TB patients registered at baseline, 650 (81.2%) were tracked. Ninety-one (11.4%) patients died and 59 (7.4%) were lost to follow-up. The rates of remaining non-smoking after 5 years were 82.0%, 63.0%, 49.6%, 43.5% and 30.0%, respectively for non-smokers, ex-smokers, current smokers who received cessation intervention, recent quitters, and current smokers not on a cessation intervention. The odds of smoking relapse were significantly higher for those aged ≥65 years (p=0.003) and registered in Xingguo County (p=0.025).

**CONCLUSIONS:**

Findings from this study confirmed that non-smokers, ex-smokers and current smokers who received cessation intervention at baseline maintained higher non-smoking rates compared with those who did not receive the intervention. To prevent relapse, intensive cessation support should be given to TB patients aged ≥65 years. TB programme managers need to ensure integration and provision of smoking cessation advice and smoke-free policy in routine TB services.

## INTRODUCTION

Although the estimated adult smoking rate in 126 countries slightly decreased from 24.7% in 2005 to 22.2% in 2015, tobacco use is still a major public health problem worldwide^1,2^. Every year, more than 7 million people die from tobacco related diseases with over 80% of deaths occurring in low- and middle-income countries^2^. On average, tobacco users lose 15 years of life^3^. Similar trends are also observed in China. Smoking rate among people aged ≥15 years in 2015 was about the same as in 2010, but the absolute number of smokers increased from 358 million to 361 million^4,5^.

Tuberculosis (TB) is an airborne infectious disease caused by *Mycobacterium tuberculosis (M. tuberculosis)*. It is the leading cause of mortality from an infectious disease^6^. Despite great progress over the past decades, TB remains a major global health problem. In 2017, an estimated 10 million people fell ill with TB implying about 20 new TB patients occurring every minute^6^. About 1.3 million individuals with TB died during the same year, translating to about 5000 deaths every day^6^.

For several decades it has been known that smoking is an independently confirmed risk factor for TB infection, with progression from infection to disease and mortality^7-9^, probably due to impairment of the host immune response^10^. Smoking is also a risk factor for TB patients’ delay in accessing health services^11^. In a recent study of 16345 TB patients in Hong Kong, smoking was found to adversely affect disease severity, sputum smear conversion, treatment outcome and relapse after successful completion of anti-TB treatment^12^. A recent report confirmed that tobacco smoking is associated with increased risk of drug-resistant TB^13^. Although evidence has strongly highlighted the significant association between tobacco use and TB, smoking cessation is not a meaningful component of National TB Programs (NTP) in most low- and middle-income countries, and health provider based smoking cessation services are urgently needed^14-17^. The World Health Organization (WHO) End TB Strategy calls for global action on co-management of TB and smoking and other comorbidities as part of basic clinical management^18^. To meet the needs at operational level, the International Union Against Tuberculosis and Lung Disease (The Union) published a guideline on tobacco cessation interventions for TB patients^19^. The approach recommended in the guideline was implemented in two counties of China and achieved a 66.7% cessation rate by the end of a 6-month anti-TB treatment^20^, but has yet to be implemented nationwide as the government needs to first ensure a sustained outcome beyond the initial intervention.

However, we did not study long-term outcomes of this smoking cessation intervention. Such information would be useful not only to help the government to scale up the intervention but also determine who are particularly at risk of a smoking relapse so that this can be prevented in the future. We therefore conducted a follow-up study to trace the registered TB patients and determine in relation to their baseline smoking status whether: 1) the non-smokers, ex-smokers and recent quitters remained non-smoking, 2) the current smokers who participated in the cessation intervention program remained at the same quitting rate as at the end of the intervention program, 3) the current smokers at the time of TB registration who did not participate in the cessation intervention program continued their daily smoking, and 4) certain baseline characteristics were associated with smoking relapse.

## METHODS

### Design and setting

**Figure 1:**
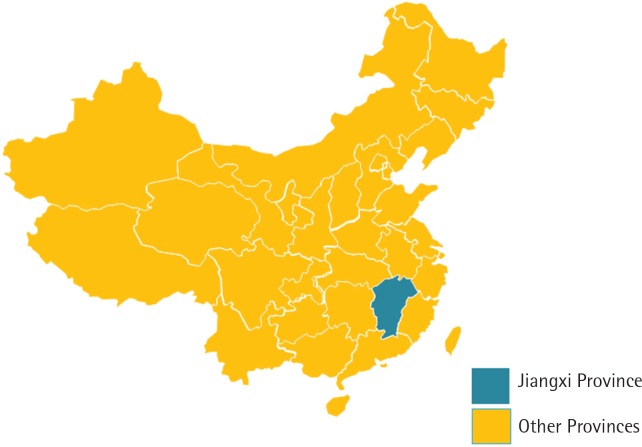
Location of Jiangxi Province in China

This was a prospective longitudinal study. The study was carried out in Ningdu and Xingguo Counties in Jiangxi Province (Figure 1). The counties are located in a mountainous area, about 150 km from a nearby city. In 2010, the estimated total population of the two counties was 1.67 million.

### Study population

In total, 800 TB patients were consecutively registered at Ningdu and Xingguo County TB dispensaries beginning March 2010 and completing their anti-TB treatment by November 2011. Over 80% were rural farmers, widely spread among 29 townships and 235 villages.

### TB diagnosis and patient management

All TB patients were diagnosed, registered, treated and managed according to the national guidelines of the China NTP^21^. Treatment regimens and anti-TB drug formulations were administered in accordance with those recommended by WHO and in line with the guidelines^22^. Directly observed therapy (DOT) was provided by rural doctors/other medical staff, family members or others according to the local practice.

### Definition of smoking status

The following operational definitions were used in this study:

*Non-smoker:* a person who had never smoked as many as 20 packs of cigarettes in his/her lifetime or one cigarette per day for at least one year.

*Ex-smoker:* a person who was previously a smoker, but had completely quit smoking for at least 90 days before the study.

*Recent quitter:* a person who had quit smoking for ≥7 days but <90 days before his/her TB registration.

*Current smoker:* a person who had ever smoked as many as 20 packs of cigarettes in his/her lifetime or one cigarette per day for at least one year, and was smoking daily or occasionally at the time of his/her TB registration.

*Smoking relapse:* For a non-smoker, ex-smoker, recent quitter or current smoker who had quit for ≥7 days at any time before the assessment, but became current smoker again at the time of the assessment.

### Cessation intervention

Details of the cessation intervention are published elsewhere^20^. A one-day training course was conducted for the health workers at the county TB dispensary. Content of the training included: orientation of The Union guide, linkage between tobacco and TB, assessing smoking status, brief advice on quitting smoking, recording, and reporting. The trained health workers recorded the patients’ smoking status immediately after their TB registration. All current smokers were encouraged to quit. Finally, current smokers who were willing to quit were recruited into a cessation intervention program. General information on the harmful health effects of tobacco use and specific information on smoking and TB were provided. The same message was re-emphasized at every visit during the entire course of anti-TB treatment.

### Tracing the registered TB patients

Records of the registered TB patients with their home addresses and telephone numbers were kept in the county TB dispensaries. A workshop was held to discuss and agree on how to effectively trace the TB patients as well as record and report their current smoking status. Final lists of TB patients by township were developed and handed over to the respective township disease control staff who convened similar meetings for rural doctors who in turn traced and visited the registered TB patients in various villages from March to May 2017.

### Data collection and analysis

Individual patient data at baseline were entered into a database that was kept in the China Office of The Union. The follow-up data were reviewed by the TB dispensary staff and then entered and analyzed by the principal investigator. Categorical comparisons of various current smoking statuses between TB patients originally classified as non-smokers, ex-smokers, recent quitters and current smokers were carried out using a chi-squared test. Relationships between relapse or continuing smoking and associated exposure variables were evaluated with odds ratios (OR) and 95% confidence intervals using logistic regression. Levels of significance were set at 5%. Variables with unadjusted ORs with p<0.05 were included in a multivariate logistic regression model.

### Ethics approval

The research was approved by the health authorities in the implementing sites and the Ethics Advisory Group of the International Union Against Tuberculosis and Lung Disease, Paris, France (EAG number: 7/17).

## RESULTS

### Baseline characteristics at registration

Of the 800 TB patients, 572 (71.5%) were male, of age 14 to 92 years with median age 46.5 years (IQR 33.0, 60.0). They consisted of 271 (33.9%) smear-positive pulmonary TB, 507 (63.3%) smear-negative pulmonary TB, and 22 (2.8%) extra-pulmonary TB (EPTB) patients. Of the registered TB patients, the number of non-smokers, ex-smokers, recent quitters and current smokers were 433 (54.1%), 100 (12.5%), 23 (2.9%) and 244 (30.5%), respectively.

### Tracing the registered TB patients after 5 years

Of the 800 registered TB patients, 650 (81.2%) were found and visited, 59 (7.4%) were lost to follow-up and 91 (11.4%) had died. We found no statistically significant difference in the proportion of found and lost to follow-up between male and female patients (p=0.142), between those ≤44 years of age and ≥45 years (p=0.936), and between non-smokers and smokers (p=0.097). However, there were strong differences within the group of current smokers in terms of higher level of smoking against those with lower level of smoking (Table 1). There was also no statistically significant difference in the proportion of found and lost to follow-up between current smokers who were on cessation intervention and those not on cessation intervention (p=1.000).

**Table 1:** Results of tracing the tuberculosis patients, stratified by gender and their baseline smoking status, Jiangxi Province, China, 2017 (N=800)

*Characteristics*	*Total*	*Found and Visited n (%)*	*Lost to follow-up n (%)*	*Died n (%)*
**Age** (years)				
14–44	375	338 (90.1)	31 (8.3)	6 (1.6)
≥45	425	312 (73.4)	28 (6.6)	85 (20.0)
p	0.936			
**Gender**				
Male	572	447 (78.2)	46 (8.0)	79 (13.8)
Female	228	203 (89.0)	13 (5.7)	12 (5.3)
p	0.142			
**Smoking status**				
Non-smokers	433	372 (85.9)	35 (8.1)	26 (6.0)
Total smokers	367	278 (75.8)	24 (6.5)	65 (17.7)
p	0.097			
**Cigarette consumption** (cigarettes/day)				
1–9	28	23 (82.1)	2 (7.2)	3 (10.7)
p	0.567			
10–29	232	180 (77.6)	13 (5.6)	39 (16.8)
p	<0.0001			
≥30	102	70 (68.6)	9 (8.8)	23 (22.5)
p	<0.0001			

### Smoking status after 5 years

Table 2 shows smoking status after 5 years stratified by a person’s baseline smoking status. The non-smokers remained most frequently as non-smokers (82.0%) followed by ex-smokers (63.0%), current smokers in cessation program (49.6%), recent quitters (43.5%), and current smokers who did not participate in a cessation program (30.0%). Proportions of TB patients who remained smokers or non-smokers were significantly different (p<0.001) among the five categories of the baseline smoking status (Table 3). Baseline characteristics of TB patients with smoking relapse in relation to their age, cigarette consumption, disease severity, patient’s TB management, and residential area are shown in Table 4. There were no statistically significant differences in these characteristics regarding the number of cigarettes smoked daily, number of lung parenchyma involved, their DOT providers, as well as treatment outcome. However, the odds of a TB patient with smoking relapse were significantly higher in those ≥65 years of age and in those residing in Xingguo County where the patients were living and registered.

**Table 2:** Proportion of the tuberculosis patients in various smoking status at baseline and at follow-up after 5 years, Jiangxi Province, China (N=800)

*Baseline status*	*Time*	*TB patients, n (%), who were in each status (cigarettes/day)*					*Died*	*Lost to follow-up*
		*Total*	*0*	*1–9*	*10–19*	*≥20*		
Non-smoker	At registration	433	433 (100.0)	0 (0)	0 (0)	0 (0)	0 (0)	0 (0)
	After 5 years	433	355 (82.0)	9 (2.1)	5 (1.1)	3 (0.7)	26 (6.0)	35 (8.1)
Ex-smoker	At registration	100	100 (100.0)	0 (0)	0 (0)	0 (0)	0 (0)	0 (0)
	After 5 years	100	63 (63.0)	7 (7.0)	5 (5.0)	1 (1.0)	21 (21.0)	3 (3.0)
Recent quitter	At registration	23	23 (100.0)	0 (0)	0 (0)	0 (0)	0 (0)	0 (0)
	After 5 years	23	10 (43.5)	2 (8.7)	1 (4.3)	1 (4.4)	7 (30.4)	2 (8.7)
Current smoker on cessation	At registration	234	0 (0)	16 (6.8)	28 (12.0)	190 (81.2)	0 (0)	0 (0)
	After 5 years	234	116 (49.6)	19 (8.1)	24 (10.3)	20 (8.5)	36 (15.4)	19 (8.1)
Current smoker not on cessation	At registration	10	0 (0)	0 (0)	3 (30.0)	7 (70.0)	0 (0)	0 (0)
	After 5 years	10	3 (30.0)	0 (0)	5 (50.0)	1 (10.0)	1 (10.0)	0 (0)

**Table 3:** Summary of smoking status of tuberculosis patients at follow-up after 5 years, stratified by their baseline smoking status, Jiangxi Province, China, 2011-2016

	*Non-smoker*	*Ex-smoker*	*Recent quitter*	*Current smoker on cessation program*	*Current smoker not on cessation program*
**Total at baseline**	433	100	23	234	10
**At follow-up after 5 years**					
Remained non-smoking, n (%)	355 (82.0)	63 (63.0)	10 (43.5)	116 (49.6)	3 (30.0)
Continuing/back to smoking, n (%)	17 (3.9)	13 (13.0)	4 (17.4)	63 (26.9)	6 (60.0)
χ^2^	104.239				
p	<0.001				

**Table 4:** Baseline characteristics of tuberculosis patients in relation to smoking relapse or continuing smoking, Jiangxi Province, China

*Characteristics*	*Categories*	*Total*	*Relapse or continuing smoking n (%)*	*Univariate regression OR ( 95% CI)*	*Multivariate adjusted regression OR ( 95% CI)*	*p*
**Age** (years) (n=800)						
	14–24	111	4 (3.6)	Reference		
	25–44	264	33 (12.5)	3.82 (1.32–11.06)	0.46 (0.14–1.49)	0.194
	45–64	286	55 (19.2)	6.37 (2.25–18.03)	1.67 (0.82–3.42)	0.161
	≥65	139	11 (7.9)	2.30 (0.71–7.43)	2.84 (1.43–5.64)	0.003
**Cigarettes/day smoked** (n=367)						
	1–9	28	5 (17.9)	Reference		
	10–29	232	50 (21.6)	1.26 (0.46–3.49)		
	≥30	102	31 (30.4)	2.01 (0.70–5.79)		
	Unknown	5				
**Lung parenchyma involved** (n=745)						
	1	203	29 (14.3)	Reference		
	≥2	542	64 (11.8)	0.80 (0.50–1.29)		
	Unknown	55				
**DOT** (n=800)						
	1^a^	531	74 (13.9)	Reference		
	2^b^	85	8 (9.4)	0.64 (0.30–1.38)		
	3^c^	184	21 (11.4)	0.81 (0.48–1.33)		
**Treatment outcome** (n=793)						
	1^d^	634	87 (13.7)	Reference		
	2^e^	159	16 (10.1)	0.70 (0.40–1.24)		
	Unknown	7				
**Counties** (n=800)						
	Ningdu	388	39 (10.1)	Reference		
	Xingguo	412	64 (15.5)	1.65 (1.08–2.52)	1.64 (1.06–2.52)	0.025

Hosmer-Lemeshow goodness of fit: χ^2^= 5.411, p=0.368, of the logistic regression model.

a Full course DOT;

b DOT provided during intensive treatment period only;

c Selfadministered therapy;

d Cured and treatment completed;

e Failure or died or lost or transferred out or unknown.

DOT: directly observed therapy.

## DISCUSSION

There are only two studies that assessed smoking cessation effect at 6 months after completion of anti-TB treatment^23^. To our knowledge, this is the first study to assess long-term changes of smoking status among TB patients. The main findings of this study are that non-smokers at the time of TB registration remained most frequently non-smokers. Among current smokers at the baseline and who participated in the cessation intervention, about one-quarter had restarted smoking after completion of TB treatment compared with 60% of those who did not benefit from the intervention. This possibly indicates a lasting outcome of the simple cessation intervention delivered by the health care providers.

Twenty-six (6.0%) non-smokers died during the 5 years. The death rate was much lower than that in the entire cohort (91, 11.4%), and greatly lower than that in smoking groups (65, 17.7%), for the same period. Death rate increased dramatically with an increasing number of cigarettes smoked daily. Our finding echoes previous research findings^2,15,24^ and indicates an urgent need to integrate smoking cessation into TB services^9^. At baseline, 33.5% of the smokers had completely quit smoking or were in the process of quitting before the cessation intervention; probably due to improved awareness of tobacco harm as a result of health promotion programs – a point that requires further investigation^20^.

As in other low- and middle-income countries, professional smoking cessation services are not available within routine TB services in China; but 95.9% of the current smokers were willing to quit and 66.7% quit smoking by the end of the anti-TB treatment as a result of brief advice by health care providers^20^. This again demonstrates that being ill with TB was the primary reason for attempting to quit and this may be an opportune time to provide information on tobacco harm and smoking cessation by TB clinicians^14^. A study on cessation in smokers suspected to have TB also found that confirmed TB diagnosis was a significant predictor of abstinence compared with those of excluded TB diagnosis^17^. However, only relying on TB diagnosis is not enough to achieve sufficient abstinence. Some studies observed that patients who received the integrated cessation intervention had significantly a higher rate of success in quitting smoking compared with those who received the conventional TB treatment alone^25,26^. We observed a similar finding in our study: among current smokers who did not participate in the cessation intervention, only 30.0% quit smoking in the same period on their own accord as a result of missed opportunity to receive comprehensive tobacco harm and cessation advice. The long-term results might demonstrate again the effectiveness of the cessation intervention offered by public TB dispensary staff. A recent study on extending a similar intervention to TB clinics operated by non-government organizations in India showed that the intervention was feasible^27^. These results suggest integration of smoking cessation intervention into routine TB services nationwide.

A small-scale study in Indonesia found that most patients quit smoking during anti-TB treatment, but over one-third relapsed at 6 months post anti-TB treatment^28^. Our findings confirm that 49.6% of the current smokers who attended the smoking cessation intervention remained non-smoking 5 years later suggesting that only 17.1% of those who quit tobacco use at the end of TB treatment relapsed during the 5 years. Reasons for this low relapse rate needs to be further studied, a reason could be improved access to health promotion programs offered by health institutions, including messages on harmful effects of cigarette smoking^29^. Looking at the 63 patients in the intervention group who resumed or continued smoking, most did so at low (1–9 cigarettes/day) to moderate (10–19 cigarettes/day) consumption level. Only 20 (31.7%) patients became heavy smokers (≥20 cigarettes/day) and this differed greatly compared with the baseline when more than 80% were heavy smokers. A similar finding was also reported in a study conducted in Indonesia^30^.

The fact that 13.0% of the ex-smokers and 17.4% of the recent quitters resumed smoking requires attention. This points to the need of extending tobacco cessation intervention to former smokers within a TB program. Another intriguing and unexpected finding, requiring further study, was the observation that 3.9% of non-smokers at baseline became smokers 5 years later. However, we do not know if they started smoking at the end of anti-TB treatment or at another time after completing the treatment. A further study is necessary to understand which patients were in this group. Some previous reports indicated that less educated patients had less access to health promotion, and, therefore, may have been less aware of the implications of tobacco use^3,31^.

This study identified certain baseline characteristics associated with smoking relapse. The relapse was not strongly associated with severity of TB disease in terms of lung parenchyma involvement determined by a chest radiography, various DOT providers, and treatment outcomes. Similar findings were also observed in the baseline factors related to abstinence at the end of anti-TB treatment^20^. For factors related to patient’s age and smoking history, we found that being ≥65 years of age was an independently confirmed risk factor for relapse. The effect of old age on smoking relapse would potentially be affected by economic status or educational level^3,31^. Less educated patients may not understand messages on cessation intervention offered by their health service providers. They may perceive that smoking is not appropriate during their disease and medication, but that they can smoke again when the treatment is over and physically felt better. The age of 65 years and above as a risk factor may also represent accumulated factors of relapse or continuing smoking, for example, being a long-time smoker or having smoked a higher number of cigarettes per day, representing addiction-related barriers^32^.

An interesting finding was that residing in Xingguo County is a risk factor for smoking relapse and this requires further investigation. In Ningdu County, there were no health workers who smoked and a smoke-free policy was implemented at the TB dispensary. However, in Xingguo County, some health workers smoked and patients’ attempts to quit smoking might have been undermined by observing a doctor smoking and exposure to tobacco smoke at the TB clinic^14,33^.

### Limitations

This study has some limitations. First, the individual patient data were collected only once after completion of their anti-TB treatment, and it follows that this design may not be able to appropriately answer the overall research question on the long-lasting benefit of the intervention. Second, the smoking status at the TB registration and at follow-up was self-reported and not confirmed through biochemical validation. Previous research has confirmed the reliability of using self-reports via plasma cotinine analysis^34^, but a recent report questioned the accuracy of self-reported smoking status^35^. There could have been some reporting bias in our study. Third, almost one in five patients could not be tracked (died or lost to follow-up) and their smoking status at 5 years after completion of anti-TB treatment remained unknown. Fourth, we did not collect data on patient’s economic status, educational background and co-morbid condition, factors that might have impacted on smoking status. Last, the number of current smokers who did not receive the intervention was small, and this may have diminished the power of comparison.

Our study aimed to assess long-term changes among TB patients with different smoking status and baseline characteristics at the time of TB registration. A large number of TB patients with different smoking behaviors were consecutively recruited in a routine program setting and were then followed up at 5 years after the completion of anti-TB treatment.

## CONCLUSIONS

This study revealed interesting findings on patients’ smoking status 5 years after completion of anti-TB treatment. Current smokers at the baseline who attended the intervention maintained higher rate of non-smoking compared with those who did not benefit from the intervention. This finding appears to support the long-term effectiveness of this simple cessation intervention delivered by TB clinicians. To prevent relapses, intensive support on smoking cessation should be given to those aged ≥65 years. Our findings suggest an urgent need to include smoking cessation and a smoke-free health facility policy as an integral part of TB services.
